# Follow-Up Imaging: Molecular Imaging is Likely Best as a Single Modality, but Multimodality Imaging is the Future

**DOI:** 10.3389/fneur.2015.00074

**Published:** 2015-04-24

**Authors:** Stephen Chiang

**Affiliations:** ^1^Radiology-Nuclear Medicine, Houston Methodist Hospital, Houston, TX, USA

**Keywords:** glioma, imaging, functional, positron emission tomography, single photon emission computed tomography, tracer

## Background

The distinction between treatment effect and recurrent brain malignancy has been a controversial topic for quite some time, with various proponents and opponents for the various cross-sectional neuroimaging modalities. The goal of this manuscript is to provide the latest information on positron emission tomography (PET) molecular imaging, offer other options within the realm of molecular imaging, and to let the reader’s ultimate decision regarding follow-up imaging be made on a case-by-case basis based upon available resources and user experience.

Delineation between treatment effect and tumor recurrence is an exceedingly important decision in the era of more focused, targeted therapy, whether anti-angiogenic, immunologic, or radiotherapy. The consequences of an incorrect assessment are obvious, leading to unnecessary treatment and morbidity, delayed treatment, or shortened survival. Various modalities have been proposed to follow such lesions after initial treatment: MRI, MRS, brain PET, and brain single photon emission computed tomography (SPECT). Only molecular imaging (PET and SPECT) will be discussed in this article.

Molecular imaging is distinguished from anatomical imaging in that it requires intact intra- and extra-cellular architecture, whereas anatomical imaging only requires an intact structure. Cancer begins at a cellular level, and thusly, microscopic changes precede macroscopic changes. No imaging modality can replace a microscopic diagnosis, but current technology attempts to model a microscopic process non-invasively. This has inherent implications of:

1. A radiographically negative scan cannot exclude microscopic disease.
2. Molecular imaging depends on specific substrate metabolism by tumors. If the particular tumor cannot metabolize or concentrate the substrate, false negative scans will occur.
3. False positive scans can occur when substrates are metabolized or concentrated by cells related to non-neoplastic processes, such as inflammation or infection.

These implications lead to the paradigm of follow up imaging after initial diagnosis and treatment: how to separate cases of residual/recurrent disease from treatment response. The optimal imaging algorithm has not been clearly delineated.

## Molecular Imaging Modalities

### Brain PET/CT

Positron emission tomography with or without inline computed tomography (CT) imaging has largely overtaken clinical SPECT imaging of the brain for functional and oncological purposes. The increased resolution of PET (3 mm theoretical) and shorter acquisition times (10 min) have significant advantages over SPECT imaging. At our institution, brain PET has largely replaced brain SPECT for both oncologic and neurologic indications. Previously, the number of PET cameras limited the availability to PET imaging, but currently, most tertiary medical centers have access to PET imaging (1). The number of installed PET/CT scanners and procedures also appears to have plateaued, however.

Positron emitting isotopes are limited in the United States, as only 18-F-fluoro-de-oxy-glucose (FDG), 11-C-choline, and 18-F-sodium fluoride (NaF) are currently approved by the FDA for routine clinical use. Sodium fluoride is a bone imaging agent, and 11-C-choline is only approved for prostate cancer imaging in the United States. Promising agents include O-2-18-F-flourorethyl-l-tyrosine (FET), 18-F-fluoro-dopamine (F-DOPA), 18-F-flouro-thymidine (FLT), and 11-C-methionine (MET); however, these are only approved under an Investigational New Drug (IND) application in the United States and are not commercially available for routine clinical use outside of a research protocol. The main topic of this article will be the use of 18-F-FDG-PET/CT.

Flouro-deoxy-glucose is structurally very similar to glucose, with the number 2 hydroxyl group substituted with F-18. It becomes a charged particle and is excreted mainly via the kidneys. However, tumor cells are unable to distinguish FDG from glucose and transport it through the cell membrane into the intracellular space, where it is phosphorylated by hexokinase into FDG-6-phosphate. Due to the polarity as well as faulty cellular enzyme machinery, FDG-6-phosphate becomes trapped within cancer cells and undergoes positron emission radioactive decay, thus localizing its distribution within the body and organs, specifically the brain in this discussion. Hence, cancer cells will concentrate FDG, although to varying degrees, often related to the degree of de-differentiation of the tumor. Increasing levels of FDG uptake associated with higher grades of gliomas has been well documented (2–4). However, background uptake of FDG by normal brain cells is quite high, leading to decreased target to background ratios. This leads to lowered overall sensitivity for FDG-PET imaging compared to a radiotracer, which is not normally metabolized by the brain, e.g., tyrosine, choline, thymidine, methionine, or dopamine. As a result, there is significant promise in these tracers, but they are not available for routine use in the US at this time. In addition, despite the increased uptake of FDG (or any other metabolic marker) within primary tumor cells, other types of metabolically active cells may concentrate the tracer, namely inflammatory cells such as neutrophils and macrophages (5). This confounds the interpretation of subsequent PET/CT studies both during and after treatment for gliomas, since both medication and radiation can induce inflammation to varying degrees and at different times. Hence, follow-up FDG-PET/CT imaging is less accurate than imaging at initial diagnosis for tumor detection. However, PET imaging has a clear advantage in that individual lesion tracer uptake can be quantified with the SUV, which is a reproducible measurement of the degree of uptake within a given lesion. A volumetric total lesion uptake can also be calculated in cases of significant disease burden. There has been considerable discussion and controversy as to the utility of FDG-PET/CT imaging in the setting of routine follow-up during and after treatment for malignant gliomas with conflicting data. The majority of false positive studies relate to underlying inflammation, with the majority of false negative studies due to small lesion size and low lesion grade (4). Follow-up imaging time after therapy is also controversial, with too early imaging potentially inaccurate due to acute inflammation, and too late imaging potentially missing opportunity for shifting the treatment scheme. Serial imaging is also useful because of the reproducibility of PET results. The trend of lesion uptake may be just as important as the absolute uptake. Benign uptake would be expected to be stable, over time, despite the absolute value. Malignant uptake would be expected to increase with time. Given comparable sensitivity of PET imaging (up to 77% using FDG-PET and 92% using 11-C-choline PET) with contrast-enhanced MRI (up to 87%) (6–8), it is the author’s opinion that while FDG-PET/CT imaging is the best choice at this time, given its relatively widespread availability, the future of neuroimaging is imaging with other tracers combined with MRI. More and more studies are showing better agreement between modalities when results are combined, rather than independently analyzed (4). 11-C-choline, in particular, shows promise (9), but is limited by its short half life of 20 min. 18-F-FET imaging shows greater promise, particularly with anti-angiogenic therapy (10–12), but is not approved for clinical use in the United States.

### Brain SPECT

Single photon emission computed tomography (SPECT) has been utilized for several decades in nuclear medicine imaging. It allows for tomographic rather than planar images and improves diagnostic accuracy. It, however, has limitations of spatial resolution and long imaging times. Spatial resolution is approximately 1 cm under ideal conditions and imaging times can be up to 45 min per acquisition. Both of these can limit its usefulness in the setting of distinguishing between tumor recurrence and post-treatment changes. However, for facilities without access to PET imaging, brain SPECT can still provide useful information, as SPECT gamma cameras are more widely accessible than PET scanners. Currently, the only clinically available brain SPECT agent for use in this setting in the US is Thallium-201 chloride. Previous studies have suggested relative robust accuracy with Th-201 (13); however, its popularity has shifted, due to increasing availability of 18-F-FDG. However, the concepts are the same, if the reader chooses to utilize Thallium brain SPECT imaging. In our experience, brain SPECT imaging is inferior to brain PET/CT imaging due to the slower throughput and inability to quantify changes that occur with time or to quantify individual findings seen on a single scan. The reader is left to subjectively describe changes and relative uptake, which often makes interpretation difficult. But SPECT imaging remains a viable option for facilities without access to PET.

## Conclusion

Molecular imaging in the setting of detecting recurrent disease and delineating treatment effect is an evolving topic, with multiple promising, newer agents being evaluated, particularly, PET tracers. The future of imaging in this setting is likely going to be a combination of multiple modalities with possibly multiple radiotracers. Serial imaging is likely to be important as well. Further research is underway to define the optimal imaging protocols. Ultimately, at this time, determining the most accurate modality is up to the reader, who must determine the best compromise, given what resources are available and his or her comfort level in interpreting the radiographic findings, but the author’s opinion is that molecular imaging is the best single imaging modality at this time, with combined multimodality imaging soon to become the top choice, particularly when radiotracers only currently used for research become available for widespread clinical use.

## Conflict of Interest Statement

The author declares that the research was conducted in the absence of any commercial or financial relationships that could be construed as a potential conflict of interest. The Guest Associate Editor Pamela Zyman New declares that, despite being affiliated to the same institution as author Stephen Chiang, the review process was handled objectively and no conflict of interest exists.
